# Head and Neck Myxoma Presenting as Isolated Laryngeal Polyp

**DOI:** 10.1155/2018/6868737

**Published:** 2018-06-10

**Authors:** Smriti Panda, Rajeev Kumar, Vikram Raj Gopinath, Prem Sagar

**Affiliations:** ^1^Department of Otorhinolaryngology and Head and Neck Surgery, All India Institute of Medical Sciences, New Delhi, India; ^2^Department of Pathology, All India Institute of Medical Sciences, New Delhi, India

## Abstract

Myxoma is a benign tumour with a propensity for local infiltration and recurrence. Laryngeal myxoma presents as a submucosal polyp. Being an uncommon tumour and mimicking vocal cord polyp, only anecdotal evidence is available in the literature. The literature was reviewed from 1986 onwards using the keywords “myxoma” and “larynx.” The databases used were PubMed, Google Scholar, Scopus, and Web of Science. Along with this, we also report our case of vocal fold myxoma. We found a total of 19 studies reporting laryngeal myxoma. Laryngeal myxoma typically affects males in the 6th decade with a history of smoking. Unlike myxomas originating outside the larynx, recurrence is not widely described, and microlaryngeal surgery will usually suffice. Laryngeal myxomas should definitely be kept in the list of differential diagnosis when dealing with a benign-looking vocal fold lesion.

## 1. Introduction

Myxomas are a rare benign myxoid neoplasm of mesenchymal origin. They are a heterogeneous group of soft tissue neoplasms with a variable degree of invasiveness, ranging from benign to highly aggressive forms [1]. Although benign, they are known to be locally infiltrative in nature with a tendency to recur if not excised with margins [2]. Myxomas of head and neck region are rare tumours with the larynx being a less commonly affected site. Clinical presentation of laryngeal myxomas is very much similar to common benign mucosal fold disorders like laryngeal polyp or cyst. Clinically, laryngeal myxomas are indistinguishable from a laryngeal polyp. They are diagnosed on histology supplemented with immunohistochemistry.

## 2. Case Report

A 53-year-old male presented with hoarseness of 12-year duration. He gave no history of breathing or swallowing difficulty. On enquiring further, he had complaints related to gastric acid reflux. He was a smoker but had quit smoking 6 months back. He is a politician with a history of voice abuse. On flexible fibreoptic evaluation, there was a 0.5 cm polypoidal, cystic mass pedicled on the medial free edge of the middle 1/3 of the right true vocal fold. There was no abnormality of vocal fold mobility. Rest of the ENT examination was normal.

Based on a history of long-standing hoarseness, voice abuse, and presence of a solitary polypoidal lesion over the true vocal fold, a preoperative diagnosis of a laryngeal polyp was made. No preoperative radiology was taken due to the unambiguous nature of the clinical findings. The patient was taken up for microlaryngeal surgery (MLS), and the lesion was excised with cold instruments. Postoperative period was uneventful with patient reporting near-normal voice during first follow-up after one week. Surprisingly, the postoperative histology showed features consistent with laryngeal myxoma.

**Figure 1:**
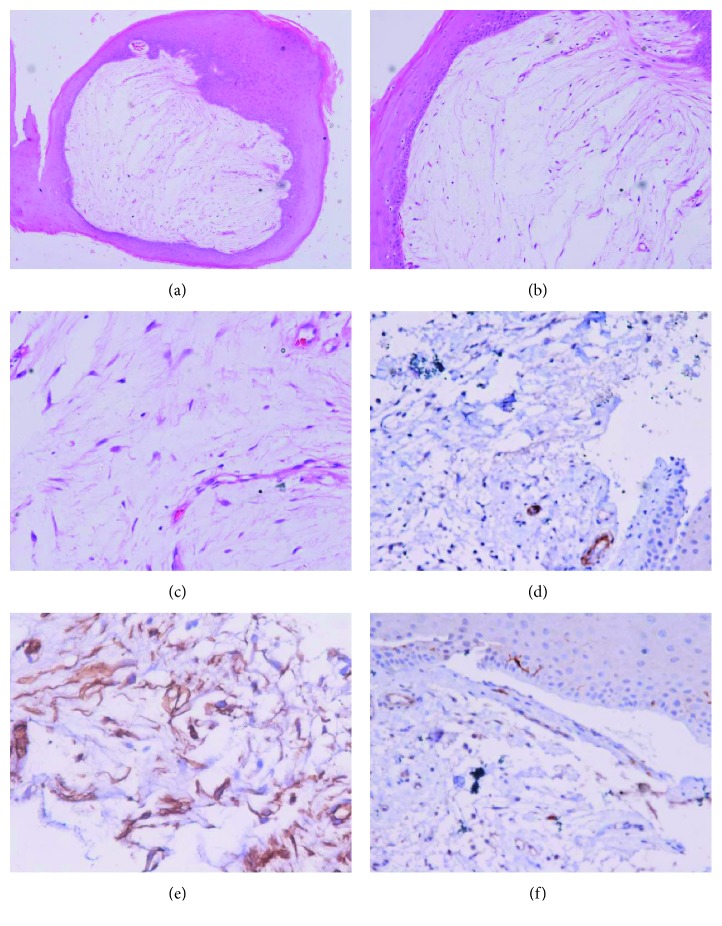
Histopathology. (a–c) A polypoidal lesion lined by hyperplastic stratified squamous epithelium. Subepithelium shows a paucicellular mesenchymal tumour comprising small, bland, spindle, or stellate-shaped cells with small and hyperchromatic nuclei and inconspicuous cytoplasm dispersed in an abundant myxoid stroma. Cellular pleomorphism, mitotic figures, and necrosis are absent. (e–f) Results of immunohistochemistry with CD34, SMA, and S-100. The tumour cells are immunonegative for CD34, smooth muscle actin (SMA), and S100 ((a) HE 40x, (b) HE 100x, (c) HE 200x, (d) CD34 200x, (e) SMA 200x, and (f) S100 200x).

On histological examination, our case showed a polypoidal tumour lined by hyperplastic stratified squamous epithelium (Figure 1(a)). A subepithelial unencapsulated lesion was noted. The latter was paucicellular formed by small, bland, spindle to stellate cells having indistinct cytoplasmic margins and hyperchromatic nuclei (Figure 1(b)). No significant atypia or mitotic activity or any necrosis was noted (Figure 1(c)). These cells were embedded within an abundant myxoid matrix. Immunohistochemically (IHC), the cells were negative for CD34, smooth muscle actin (SMA), and S100 (Figures 1(d)–1(f)). Thus, a final diagnosis of laryngeal myxoma was rendered. The absence of stromal vasculature, hemorrhage, hemosiderin-laden macrophages, and hyalinization of basement membrane helped to differentiate it from a vocal fold polyp [3].

## 3. Discussion

We did a comprehensive review of all the anecdotal cases of laryngeal myxoma reported in the literature so far which is summarized in Table 1 [3–20] after searching across PubMed, Google Scholar, Scopus, and Web of Science using the terms “Myxoma” and “laryngeal.” All articles reporting laryngeal myxoma specifying tumour location and histological feature were included.

Larynx as a site for head and neck myxoma is extremely rare. Our comprehensive literature search revealed 19 cases of laryngeal myxoma till date (Table 1). Male preponderance (M : F of 5 : 1) with a history of smoking is seen in a majority of cases reported. Our case also had both of these characteristics. Mean age at presentation was 52.16 years (range 36 to 77 years). Hoarseness was the most common presenting symptom signifying a predilection for the glottis, as seen in our case also. Dysphagia was reported in the studies by Baruah et al. and Chen et al. due to the involvement of the epiglottis [15, 20]. All authors documented the presence of a submucosal polypoidal mass ranging in size from 0.4 cm to 6.5 cm. Microlaryngeal surgery and excision by cold instruments was the preferred approach with one reported recurrence which responded to subtotal excision [4]. Tracheostomy was required preoperatively in two glottic myxomas due to airway compromise which was sorted after complete excision [5, 16]. Sena et al. utilized a transcervical route in his case where the primary one was located in the aryepiglottic fold [19].

Although we did not find any systemic association, at least three studies have revealed an association with Reinke's edema [3, 11, 16]. Possibility of Reinke's edema being a precursor lesion or sharing the same etiopathogenesis with myxoma needs to be studied in detail.

Demographically, laryngeal myxomas differ from other head and neck myxomas. A comprehensive review on head and neck myxoma by Andrews et al. [2] shows a female preponderance, with age group 18–67 years being affected. This study also quoted involvement of the paediatric age group. Laryngeal myxoma, on the contrary, seems to be a disease of males in the 5th–6th decade with a history of smoking.

Various theories for its etiopathogenesis have been put forth. Due to the abundance of mucinous matrix and glycosaminoglycans, fibroblast immaturity has been cited as a factor [21]. Another theory traces its origin to odontogenic primordial mesenchyme [3]. The latter probably explains the most common location for a myxoma in head and neck, that is, maxilla. Immature fibroblasts could be responsible for the occurrence in the larynx and also the association seen with Reinke's edema. Myxomas have been reported in mucopolysaccharidosis, Carney complex, and Mazabraud syndrome [3]. Mazabraud syndrome belongs to the spectrum of fibrous dysplasias where intramuscular myxomas have been described [22]. Carney complex is an autosomal dominant disorder characterized by cardiac myxomas, cutaneous hyperpigmentation, and multiple endocrinopathies in the form of raised ACTH and growth hormone. PRKAR1 alpha is the tumour suppressor gene implicated in this syndrome.

Histopathology forms the mainstay for diagnosing this entity. The usual clinical appearance is that of a benign vocal fold lesion like an intracordal cyst, polyp, or a nodule. It is the presence of myxoid stroma and abundance of stellate cells and spindle cells that help in clinching the diagnosis. There is no general consensus regarding myxoma-specific immunohistochemistry markers. All studies (Table 1) have reported submucosal involvement, a predominance of stellate cells and spindle cells against the background of a mucinous matrix. Immunohistochemistry markers were available in three studies with no consensus regarding the significance of positivity of markers like CD34, SMA, S100, desmin, and Ki-67.

Head and neck myxomas are known for locally infiltrative nature and tendency to recur if not excised radically [2]. However, literature review suggested that microlaryngeal surgery performed from the point of view of a vocal fold polyp or cyst will generally suffice as it is evident by only one recurrence reported so far and long disease free intervals in other patients.

## 4. Conclusion

Laryngeal myxomas should definitely be kept in the list of differential diagnosis when dealing with a benign looking vocal fold lesion. Patients need to be kept on close follow-up if excision has been suboptimal due to the absence of capsule and locally infiltrative nature.

## Figures and Tables

**Table 1:** Systematic review of all cases of laryngeal myxoma.

Author (year)	Number of cases	Age/sex	Presentation	Addiction	Association	Site	Size	Approach	Follow-up	Histology	Need for tracheostomy
Tang et al. (2015) [4]	1	ND	Dysphonia	ND	—	Glottis	ND	Two approaches	?Recurrence		—
Ritchie et al. (2015) [3]	1	77/M	Hoarseness 6 months	Smoker	Reinke's edema	Glottis	<1 cm	MLS	3 months, NED	Stellate cell, spindle cell, and mucinous matrix. CD 34−, S100+, and SMA+	—
Singh et al. (2014) [5]	1	65/M	Hoarseness 4 months and dyspnoea 1 week	ND	—	Glottis	17 × 12 mm	MLS	8 months, NED	-do-	Tracheostomy
										No IHC	
Shah et al. (2014) [6]	1	50/M	Hoarseness 2 months	ND	—	Glottis	—	MLS	No f/u	-do-	—
										No IHC	
Garca et al. (2013) [7]	1	61/M	Hoarseness 3 months	Smoker	—	Glottis	0.5 × 0.5 cm	MLS	No f/u	-do-	—
										CD 34−, S100−, desmin−, and SMA−	
Kanlıada et al. (2012) [8]	1	42/f	Hoarseness 3 months	—	—	Glottis	4 mm	MLS	12 months, NED	-do-	—
										No IHC	
Nakamura et al. (2008) [9]	1	74/f	Hoarseness	Alcohol	—	Glottis	4 mm	MLS	NED	-do-	—
										No IHC	
Song et al. (2008) [10]	1	36/M	Hoarseness 2 months	—	—	Glottis	7 mm × 5 mm	MLS	4 months, NED	-do-	—
										No IHC	
Ali et al. (2008) [11]	1	48/F	Hoarseness childhood	Smoker	Reinke's edema	Glottis	—	MLS	—	-do-	—
										CD34+, S100−, and SMA−,Ki-67+	
Leu et al. (2007) [12]	1	53/m	Hoarseness 2 years	—	—	Glottis	5 mm	MLS	—	-do-	—
										No IHC	
Kim et al. (2007) [13]	2	62/m	Hoarseness 5 years	Smoker	—	Glottis	1.5 × 0.8 × 0.4 cm	MLS	8 years, NED	-do-	—
										No IHC	
		52/M	Hoarseness and dyspnoea 3 weeks	Smoker	—	Glottis	2 × 0.4 × 0.4 cm	MLS	3 years, 6 months, NED	-do-	—
										NED	
Idrees et al. (2005) [14]	1	46/M	—	Smoker	—	Glottis	8 mm	MLS	—	-do-	—
										No IHC	
Baruah et al. (2001) [15]	1	57/M	Hoarseness	—	—	AEF/epiglottis	6.5 × 5.0 × 1 cm	MLS	ND	-do-	—
										No IHC	
Kim et al. (1997) [16]	1	62/M	Dyspnoea 3 days	Smoker, alcoholic	Reinke's edema	Glottis, obstructing airway	2.5 × 2.5 × 1.5 cm	MLS	—	-do-	Tracheostomy
										No IHC	
Tsunoda et al. (1997) [17]	1	57/m	Hoarseness	Alcoholic	—	Glottis	7 × 7 × 7 mm	MLS	NED	No IHC	—
Hadley et al. (1994) [18]	1	64/M	Hoarseness 4 years	Alcoholic, smoker	—	Glottis	1 × 0.6 × 0.2 cm	MLS	18 months, NED	No IHC	—
Sena et al. (1991) [19]	1	70/M	Hoarseness	Alcoholic, smoker	—	AEF	0.5 × 0.5 × 2.5 cm	Transcervical	12 months, NED	No IHC	—
Chen and Ballecer (1986) [20]	1	37/M	Dysphonia and dysphagia	—	—	Epiglottis	5.6 × 4.3 × 2.4 cm	MLS	12 months, NED	No IHC	—
Present study (2016)	1	53/M	Hoarseness 12 years	Smoker	—	Glottis	0.5 cm	MLS	4 months, NED	Immunonegative for CD34, SMA, and S100	—

## Data Availability

The datasets generated or analysed during this study are available from the corresponding author on reasonable request.
